# Parasite Infections Influence Immunological Responses But Not Reproductive Success of Male Hellbender Salamanders (*Cryptobranchus alleganiensis*)

**DOI:** 10.1093/iob/obaf006

**Published:** 2025-04-03

**Authors:** K L Slack, J Groffen, A K Davis, W A Hopkins

**Affiliations:** Department of Fish and Wildlife Conservation, Virginia Tech, Blacksburg, VA 24061, USA; Department of Fish and Wildlife Conservation, Virginia Tech, Blacksburg, VA 24061, USA; Odum School of Ecology, University of Georgia, Athens, GA 30602, USA; Department of Fish and Wildlife Conservation, Virginia Tech, Blacksburg, VA 24061, USA

## Abstract

The emergence and spread of infectious diseases is a significant contributor to global amphibian declines, requiring increased surveillance and research. We assessed host–vector–parasite dynamics using a population of eastern hellbender salamanders (*Cryptobranchus alleganiensis*) that harbor leeches (*Placobdella appalachiensis*) that transmit endoparasitic blood parasites (*Trypanosoma* spp) to the host, with coinfection frequently occurring. We centered our study on adult males throughout their extended 8-month paternal care period because recent research indicates that nest failure caused by lack of paternal care and filial cannibalism is contributing to hellbender population declines. Recognizing the potential for parasites to modulate host physiology and behavior, we explored how infection severity influences paternal health and reproductive success. We assessed white blood cell profiles of adult male hellbenders in response to parasites, coinfection, and seasonal temperature fluctuations, while also investigating whether parasite infection or coinfection was predictive of nest success. We found that hellbenders exhibited seasonal shifts in white blood cell indices; as temperatures increased across seasons (from 5°C to 20°C), the proportion of neutrophils and eosinophils decreased (by 14% and 46%, respectively) in circulation while the proportion of lymphocytes and basophils increased (by 8% and 101%, respectively). Moreover, the proportion of neutrophil precursors increased by 80% under colder temperatures, which signifies seasonal immune cell recruitment. We demonstrated that neutrophils and eosinophils increased while lymphocytes decreased in response to leech infection. However, as leech and trypanosome infection intensity increased together, the proportion of lymphocytes increased while neutrophils and eosinophils decreased, underscoring the complex interactions between coinfection and immune responses of hellbenders that warrant future research. Despite the influence of infection and coinfection on hellbender physiology, we detected no evidence to support the hypothesis that parasites influence the likelihood of nest failure or whole-clutch filial cannibalism. In light of amphibian declines being exacerbated by climate change and disease, our study emphasizes the need to establish hematological reference values that account for physiological adaptations to seasonal fluctuations in temperature and different life history stages and to study the physiological responses of imperiled amphibian species to parasites.

## Introduction

Amphibians are among the most endangered vertebrates, with 41% of species threatened with extinction, predominantly due to climate change, habitat loss and degradation, and emerging infectious diseases (Hoffman et al. 2010; Luedtke et al. 2023). Due to their physiology and specialized habitat requirements, amphibians are particularly susceptible to subtle perturbations in the environment (Vitt et al. 1990; Wake 1991; Blaustein et al. 1994; Hopkins 2007), which makes them valuable indicators of environmental conditions (Blaustein 1994; Blaustein and Wake 1995; Hopkins 2007). Therefore, investigating aspects of amphibian physiology can provide insight into how environmental factors impact individual health and even population dynamics. However, investigations into threats to amphibians must also consider the complex, localized environmental conditions involved. For example, nearly all aspects of the physiology of wild amphibians fluctuate with environmental temperatures (da Silva et al. 2013; Herczeg et al. 2021). As such, recognizing seasonal patterns in physiological indices is essential to distinguish between natural variation and the impact of other factors such as disease.

Infectious diseases have been implicated in the decline of many amphibian populations (Daszak et al. 1999; Yap et al. 2015; Fisher and Garner 2020), with some diseases directly linked to species extinction (Daszak et al. 2003; Retallick et al. 2004; Lips et al. 2006). Parasites and pathogens can negatively influence population dynamics by directly impacting host physiology and causing disease (Davis et al. 2007; Johnson and McKenzie 2009; Bower et al. 2019), which can lead to localized mass mortalities (Davis et al. 2007; Lips et al. 2008; Price et al. 2014). But parasites can have more subtle effects on hosts that pose insidious risks to their populations. For example, among other taxa, parasites mediate deleterious host behaviors with implications for population dynamics (Haye and Ojeda 1998; Dick et al. 2010; Bunke et al. 2015). Parasite infection can also negatively affect fitness, with parasitized hosts suffering reductions in fecundity and mating success (Lehmann 1993; Hasik and Siepielski 2022). Therefore, it is essential to investigate how parasites may directly and indirectly impact wild amphibians to understand the causes of population declines.

The eastern hellbender (*Cryptobranchus alleganiensis*) is an imperiled amphibian species that is infected with multiple types of parasites and is thus well suited for studies seeking to disentangle natural fluctuations in physiological variables from those caused by parasitic infection. Hellbender populations have undergone steep declines in population abundance since the 1970s, with some declining by 70–80% (Wheeler et al. 2003; Burgmeier et al. 2011; Graham et al. 2011) and other populations being extirpated or functionally extinct (US Fish and Wildlife Service 2018). The factors causing their population declines are multifaceted, but habitat degradation and disease are among the most common factors implicated (US Fish and Wildlife Service 2018; Hopkins et al. 2023; US Fish and Wildlife Service 2024). Thus, hellbenders are a good species for investigating physiological responses to factors like parasites that can compromise amphibian health and survival.

In some streams in southwest Virginia, hellbenders are exposed to sanguivorous leeches, *Placobdella appalachiensis* (Hopkins et al. 2014), which are the vectors for a newly discovered but unnamed trypanosome species, *Trypanosoma* spp (Davis and Hopkins 2013). Available evidence suggests that both parasites, leech and trypanosome, are most prevalent in stream reaches with high density of hellbenders (Jachowski and Hopkins 2018; Bodinof Jachowski et al. 2024), demonstrating the importance of host density on the parasites’ population dynamics. The parasites are known to elicit immune responses (Hopkins et al. 2016; Bodinof Jachowski et al. 2024), cause symptoms of anemia (Slack et al. 2024), and the leech is implicated in endocrine disruption (DuRant et al. 2015) in the hosts. However, whether these parasites affect any fitness-related traits of the hosts remains unknown. Because recent investigations have revealed that high rates of nest failure caused by inadequate paternal care and whole-clutch filial cannibalism may be contributing to population declines (Hopkins et al. 2023; Brooks et al. 2024), understanding whether parasitic infection influences paternal care and resulting reproductive success is a high priority.

Shifts in proportions of various white blood cell (WBC) types in circulation can provide valuable insights into how amphibian immune systems are influenced by parasites and other extrinsic factors (Allender and Fry 2008; Campbell 2015; Forzán et al. 2017; Davis and Golladay 2019; Bain and Harr 2022; Davis and Maerz 2023). Prior hellbender studies have used WBC profiles to document the effects of parasites, which included profiles indicative of antiparasitic and inflammatory responses (Durant et al. 2015; Hopkins et al. 2016; Bodinof Jachowski et al. 2024). However, considering the ectothermic physiology of hellbenders, it is important to understand how seasonal shifts in temperature could potentially influence WBCs. For example, temperature affects the immune performance of amphibians, and prolonged exposure to cold may suppress components of the adaptive immune response (Maneiro and Carey 1997). Additionally, fluctuations in temperature could leave amphibians more susceptible to infection because of the suppressive effects of variable thermal conditions on the immune system (Raffel et al. 2006b). Thus, a more thorough understanding of seasonal variation in WBC composition in hellbenders is needed to effectively interpret their immune responses to parasites or other environmental factors.

In this study, we assessed the effects of differences in temperature due to seasonality and parasite load on WBC profiles of male hellbenders and ascertained whether parasitic infection is associated with nest success in hellbenders. We focused on males guarding their nests because the parental care period is known to be a particularly sensitive time for vertebrates that exhibit care (Smith and Wootton 1995; Harshman and Zera 2007), including hellbenders (Hopkins et al. 2023), and some blood parasites are thought to exert their greatest effect on hosts during demanding or strenuous life history stages (Nordling et al. 1998; Norte et al. 2009). Based on the known functions of WBCs in other ectothermic vertebrates (see descriptions of cell types and functions in methods), we hypothesized that hellbenders would exhibit seasonal shifts in WBC profiles correlated to environmental temperatures, prioritizing aspects of the innate immune response in colder temperatures. We predicted that the proportion of circulating lymphocytes and basophils would decrease with temperature while the proportion of eosinophils and neutrophils would increase as temperature decreases (Maneiro and Carey 1997; Raffel et al. 2006b; Bodinof Jachowski et al. 2024). We also predicted that as temperatures decrease, hellbenders would exhibit signs of immune recruitment through increased proportions of band and toxic neutrophils. We also hypothesized that parasitic infection would influence WBC profiles, resulting in an inflammatory and antiparasitic immune response. Under this hypothesis, we predicted that the proportions of eosinophils and neutrophils in circulation would increase in response to parasitic infection, while the proportion of lymphocytes and basophils would decrease as they migrate out of the vascular space into tissues (Latimer et al. 2003; Eberle and Voehringer 2016). We also predicted that the proportion of band and toxic neutrophils would increase with parasite infection intensity due to the prolonged demand for inflammatory and innate immune responses (Stacy et al. 2022). Lastly, we hypothesized that parasite infection would influence nest success. Because recent research indicates that some male hellbenders forgo paternal care and engage in whole-clutch filial cannibalism for unknown reasons (Hopkins et al. 2023; Brooks et al. 2024), we predicted that the likelihood of whole-clutch cannibalism and/or nest failure would increase as parasite infection intensity increases in male hellbenders.

## Materials and methods

### Species description

The eastern hellbender (*Cryptobranchus alleganiensis alleganiensis*) is a fully aquatic giant salamander of the family Cryptobranchidae and represents one of the oldest lineages of salamanders (Pyron and Wiens 2011). It is the heaviest amphibian in North America, with a lifespan of more than 25 years (Nickerson and Mays 1973; Taber et al. 1975), and it can weight up to 2.2 kg and grow up to 74 cm in total length (Petranka 1998). Hellbenders tend to thrive in cool, fast-flowing, oxygen-rich streams (Nickerson and Mays 1973) with low organic matter and fine sediments (Pugh et al. 2016) in areas with extensive boulder habitat and high upstream forest cover (Pugh et al. 2016; Jachowski and Hopkins 2018). As a notoriously cryptic salamander, hellbenders primarily rely on rock crevices for cover and reproduction (Nickerson and Mays 1973).

Hellbender reproductive ecology differs from most other North American salamanders. While the timing of the breeding season can vary depending on environmental factors and geographic location (Nickerson and Mays 1973), in our study, the timing of oviposition ranged from August 22 to September 22. After securing a suitable crevice under a large boulder to breed, male hellbenders engage in fully aquatic, external fertilization followed by prolonged, solitary, obligate paternal care of their eggs and larvae (Bishop 1941; Nickerson and Mays 1973; Hopkins et al. 2023). In Virginia, male hellbenders are known to stay with their young for more than 8 months (Hopkins et al. 2023). Following an ∼60-day embryonic developmental period, larvae hatch from eggs and remain in the nest with the guarding male until the following April to May (Hopkins et al. 2023).

Hellbenders in our study population are host to sanguivorous leeches (*P. appalachiensis*) and a trypanosome (*Trypanosoma* spp) that is vectored by the leech (Davis and Hopkins 2013; Hopkins et al. 2014; Jachowski and Hopkins 2018; Bodinof Jachowski et al. 2024; W. A. Hopkins et al., unpublished). These leeches are known to inhabit hellbender nest cavities and boulders used for shelter year-round and exhibit brooding and parental care behaviors in the summer months (A. Blumenthal and W. A. Hopkins, unpublished). Following the leech brooding period, the parent delivers the leech offspring to its first blood meal (A. Blumenthal and W. A. Hopkins, unpublished), the timing of which is synchronous with the beginning of the hellbender breeding season (Hopkins et al. 2023; O'Brien et al. 2024) when multiple hellbender hosts are entering nest cavities and establishing territories (O'Brien et al. 2024). Experimental infection confirmed that the leech is an effective vector for the trypanosomes (W. A. Hopkins et al., unpublished), a hematophagous endoparasite that is highly prevalent in local populations of hellbenders (DuRant et al. 2015; Hopkins et al. 2016; Bodinof Jachowski et al. 2024; Slack et al. 2024). Notably, a recent study suggests that peak trypanosome transmission occurs during the simultaneous reproductive periods of the hellbender host and leech vector (Slack et al. 2024).

### Site description

Our study was conducted in four stream reaches (each ∼100–200 m long) along an ∼13-km segment of a single stream within the Upper Tennessee River Basin (VA, USA). We selected reaches with similar site quality, upstream catchment-wide riparian forest cover (64.3–67.9%), and high population densities of healthy hellbenders that successfully reproduce annually (Jachowski and Hopkins 2018; Hopkins et al. 2023). Based on 8 years of monitoring, annual nest success in these four reaches averaged 49%, and rates of whole-clutch filial cannibalism were 14% (Hopkins et al. 2023).

Underwater artificial shelters were deployed up to 10 years prior to this study in each stream reach (Hopkins et al. 2023). The number of shelters varied by stream reach but ranged from 30 to 35 per reach. These shelters mimic cavities under large boulders that hellbenders use for year-round shelter and nesting habitat and are used by our research team to monitor population dynamics, behavioral ecology, physiology, and annual reproduction (Button et al. 2020a, b; Jachowski et al. 2020; Hopkins et al. 2023; O'Brien et al. 2024; O'Brien et al. 2025). We monitored the hourly temperature of each stream reach using U24-001 HOBO® Conductivity Data Loggers 0–10,000 µS/cm (Onset Computer Corp, Bourne, MA, USA).

### Sample collection

We captured reproductive male hellbenders nesting in our artificial shelters at four intervals across the paternal care period in 2020 and 2021. Sex was confirmed based on external cloacal morphology (Makowsky et al. 2010). We sampled each individual at oviposition (Day 0 [August 30 to September 22], *N* = 58) and attempted recapture at mid-embryonic development (∼Day 30 [October 2 to October 17], *N* = 48), larval hatching (∼Day 60 [November 5 to November 21], *N* = 44), and just prior to juvenile emergence from the nest (∼Day 200 [March 25 to April 20], *N* = 25) or until nest failure (Table 1). Our prior work demonstrated that adult male hellbenders are tolerant of repeated capture and blood collection and that repeated handling does not affect their parental care or nesting success (Hopkins et al. 2023; O'Brien et al. 2025).

**Table 1:** Characteristics of WBC parameters of adult male eastern hellbenders (*Cryptobranchus alleganiensis*) across the 8-month parental care period from a population in southwest Virginia, USA.

	Sampling day											
	Oviposition (*N* = 56)			Mid-embryonic (*N* = 48)			Hatching (*N* = 44)			Emergence (*N* = 25)		
Parameter	Uninfected (*N* = 9)	Tryp only (*N* = 23)	Coinfected (*N* = 24)	Uninfected (*N* = 5)	Tryp only (*N* = 23)	Coinfected (*N* = 20)	Uninfected (*N* = 5)	Tryp only (*N* = 25)	Coinfected (*N* = 13)	Uninfected (*N* = 3)	Tryp only (*N* = 15)	Coinfected (*N* = 7)
Neutrophils (%)	25.9 ± 2.2	26.1 ± 1.7	32.2 ± 1.7	27.3 ± 2.0	31.1 ± 1.7	32.9 ± 1.6	23.9 ± 2.8	29.6 ± 1.5	34.0 ± 2.1	27.8 ± 2.6	27.9 ± 2.1	25.2 ± 2.1
Lymphocytes (%)	61.2 ± 2.9	60.9 ± 2.0	51.9 ± 2.2	61.0 ± 1.5	54.9 ± 1.6	53.5 ± 1.9	66. ± 3.7	58.1 ± 1.8	52.7 ± 2.2	55.5 ± 5.8	57.5 ± 1.8	55.3 ± 2.4
Eosinophils (%)	5.0 ± 1.2	7.3 ± 0.9	9.9 ± 1.0	6.9 ± 1.4	9.8 ± 1.0	9.7 ± 0.7	6.5 ± 1.5	8.6 ± 0.8	9.0 ± 1.2	14.2 ± 5.5	10.4 ± 1.0	12.7 ± 1.2
Basophils (%)	7.0 ± 1.2	5.4 ± 0.6	5.4 ± 0.5	4.5 ± 0.7	3.82 ± 0.5	3.6 ± 0.5	2.3 ± 0.6	3.1 ± 0.3	3.8 ± 0.4	2.3 ± 0.2	3.9 ± 0.2	6.3 ± 1.1
Band neutrophils (%)	4.8 ± 0.9	5.3 ± 0.8	6.3 ± 1.2	5.5 ± 2.2	5.1 ± 0.8	5.6 ± 1.0	8.0 ± 4.0	8.1 ± 1.4	8.7 ± 2.0	4.3 ± 0.4	5.3 ± 0.9	8.4 ± 2.4
Toxic neutrophils (%)	14.5 ± 2.0	16.7 ± 1.5	15.8 ± 1.7	18.3 ± 2.2	17.0 ± 1.8	18.8 ± 1.8	12.6 ± 2.6	18.6 ± 1.7	18.1 ± 2.0	8.1 ± 0.5	11.9 ± 1.9	12.5 ± 1.5
N:L ratio	0.44 ± 0.05	0.46 ± 0.05	0.68 ± 0.06	0.45 ± 0.0	0.59 ± 0.05	0.65 ± 0.06	0.37 ± 0.07	0.54 ± 0.04	0.68 ± 0.07	0.51 ± 0.08	0.51 ± 0.05	0.47 ± 0.05

Arithmetic means (±1 SEM) are presented for WBC parameters in adult male hellbenders across the parental care period and infection status. Sampling day represents repeated capture intervals corresponding to offspring development in which oviposition represents nest initiation (Day ∼0), mid-embryonic represents mid-embryonic development (Day ∼30), hatching represents larval hatching (Day ∼60), and emergence represents spring larval emergence (Day ∼200). Parameters represent the proportion of each cell type out of the total counted within 50 fields of view on whole blood smears. N:L ratio represents the proportion of neutrophils divided by the proportion of lymphocytes.

At each interval we removed a male hellbender from its artificial shelter and used a heparinized syringe to obtain a baseline (within 3 min of capture) sample of whole blood (collecting an average of ∼69 μL/100 g of body mass), following the methods outlined in Hopkins and DuRant (2011). We then transported the individual in a plastic bin containing fresh stream water to the stream bank for processing. We assessed the hellbender's body condition, noted any abnormalities in physical appearance such as injury, and obtained standard body morphometrics, including snout-to-vent length (cm), total length (cm), and mass (g). We recorded the number of leeches attached and the number of leech bite wounds on each individual as an indicator of recent infection, which are easily identified (circular lesions at the detachment site that are visible on the body for several weeks) and distinct from any other markings on hellbenders. We used uniquely coded passive integrated transponder (PIT) tags that had been implanted in prior years to identify each hellbender. If individuals were newly captured in this study, we inserted a PIT tag subcutaneously along the tail's dorsolateral region, ∼5 cm posterior to the tail base, prior to release back to its artificial shelter (Hopkins and DuRant 2011).

### Blood processing

At each interval where a baseline blood sample was collected, we made duplicate blood smears with fresh whole blood using a standard two-slide wedge technique. We also injected ∼75 μL of whole blood into two heparinized capillary tubes and transported them on ice to the laboratory. Within 8 h of sample collection, we centrifuged duplicate capillary tubes for each individual at 5 *g* for 5 min. We used a Hamilton syringe to remove plasma from the upper layer of the sample, which was then archived at −80°C for companion endocrinology studies. We then removed the buffy coat, the thin white layer of WBCs on top of packed red blood cells, which is also where trypanosome parasites partition during centrifugation (Hopkins et al. 2016). We made duplicate buffy coat smears using the standard two-slide wedge technique to quantify trypanosome infection status, a technique that greatly increases the detectability of trypanosomes compared to standard whole blood smears (Hopkins et al. 2016). Buffy coat and whole blood smears were stained using Camco Quik Stain II (Cambridge Diagnostic Products, Fort Lauderdale, FL, USA) and air-dried in preparation for microscopy.

### Microscopy

**Fig. 1:**
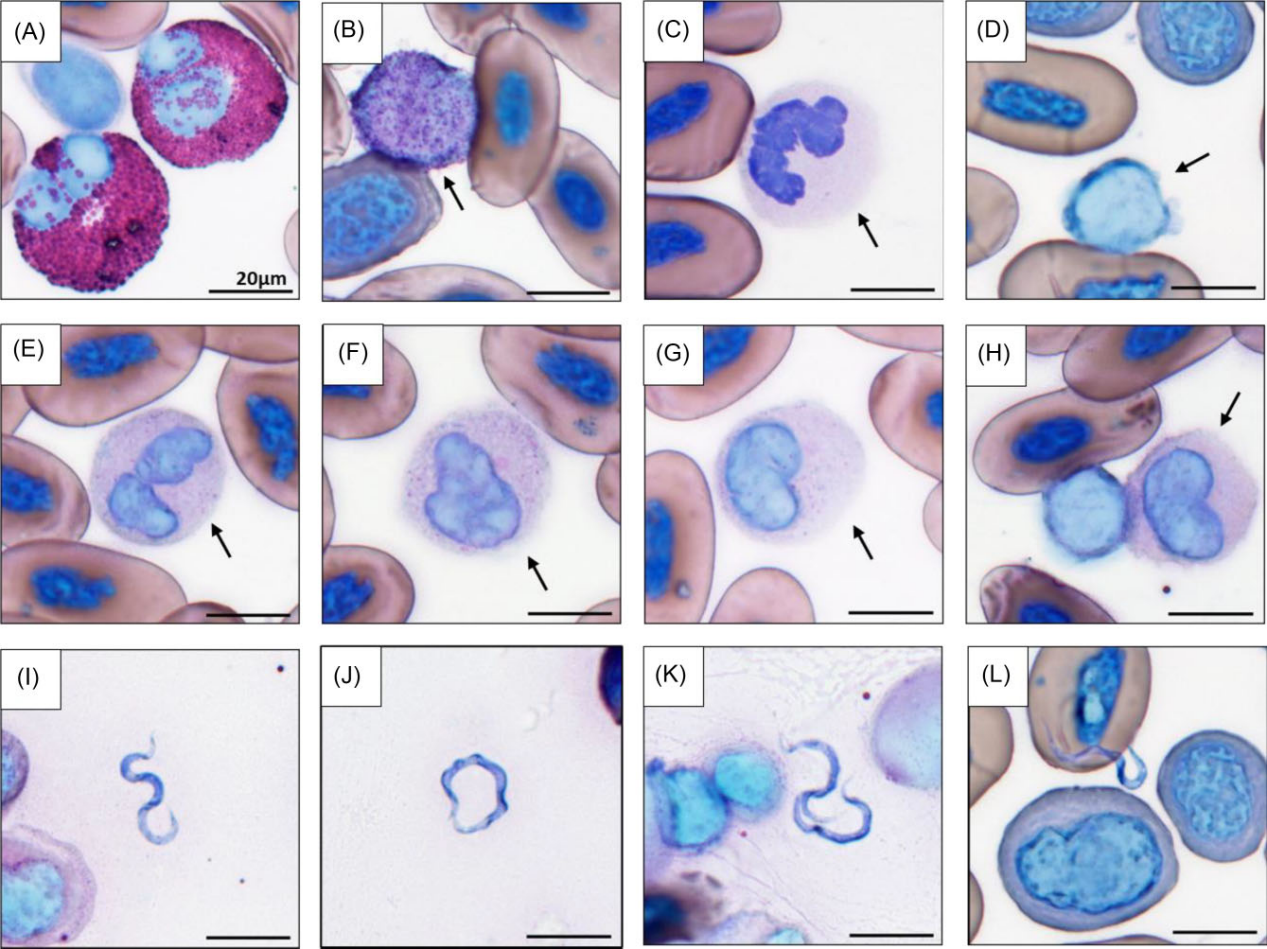
Microscopic images of hellbender WBCs and trypanosomes. Images include (**A**) two eosinophils (pink staining) alongside a thrombocyte, (**B**) a basophil (arrow) next to a polychromatic (immature) red blood cell and mature red blood cells, (**C**) a healthy mature neutrophil (arrow) next to partial mature red blood cells, (**D**) a lymphocyte (arrow) adjacent to partial mature red blood cells and polychromatic red blood cells, (**E** and **F**) mature neutrophils (arrow) with toxic granulation with partial mature red blood cells, (**G**) band neutrophil (arrow) with visible secondary granules, (**H**) band neutrophil (arrow) with visible secondary granules, alongside a lymphocyte and mature red blood cells, (**I–K**) trypanosomes from buffy coat smears, and (**L**) a trypanosome from whole blood smear alongside mature and polychromatic (young) red blood cells. Images were taken on a compound light microscope at 400× magnification. The black scale bar represents 20 µm.

For microscopy, a single observer (K. Slack) was blinded to the identity of each slide and examined 50 random fields of view in both whole blood and buffy coat smears. We used a light microscope at 400× magnification to quantify WBC differentials in whole blood smears and the number of trypanosomes in buffy coat smears. We quantified the proportion of eosinophils, basophils, neutrophils, lymphocytes, and monocytes, which based on their specific function can be informative for identifying ongoing stressors and immune activity (Davis et al. 2008; Forzán et al. 2017). Eosinophils (Fig. 1A) play a role in the inflammation process and antiparasitic defense (Latimer et al. 2003). Basophils (Fig. 1B) migrate out of circulation and into peripheral tissues during allergic reactions and parasitic infection and are also important in the inflammatory response as well as protective immunity against parasites (Eberle and Voehringer 2016). The proliferation of neutrophils (Fig. 1C) into circulation occurs in response to stress, inflammation, and infection (Latimer et al. 2003). Lymphocytes (Fig. 1D) are the predominant leukocyte type in amphibians (Bain and Harr 2022) and are involved in multiple immunological processes in which they migrate out of peripheral circulation into tissues upon stimulation (Latimer et al. 2003). Notably, the glucocorticoid stress response promotes the paired migration of neutrophils into circulation and lymphocytes out of circulation into tissues. As a result, the relationship between the two cell types is often represented as a ratio (i.e., N:L ratio) and has become a common metric used by researchers to evaluate the vertebrate stress response (Davis et al. 2008). Monocytes also play a role in mediating inflammatory responses and eliminating foreign substances (Latimer et al. 2003); however, hellbenders have very few in circulation (Hopkins et al. 2016; Bodinof Jachowski et al. 2024).

In addition to quantifying these standard types of WBCs, we went further to distinguish different types of neutrophils. We calculated the proportion of neutrophils with toxic change (hereafter referred to as toxic neutrophils; Fig. 1E and F) and neutrophil precursors (hereafter referred to as band neutrophils; Fig. 1G and H) out of the total number of circulating WBCs. Toxic neutrophils occur when a rapid increase in neutrophil demand triggers accelerated production, leading to abnormal maturation (Stacy et al. 2022). While the morphological appearance of toxic neutrophils varies across taxa, distinctive features used to recognize them include degranulation, cytoplasmic vacuolation or foaminess, and variation in the shape and color of cytoplasm granules (Fig. 1E and F; Stacy et al. 2022). Increased proportions of band neutrophils occur when inflammatory demand surpasses neutrophil storage capacity. Characteristics used to identify band neutrophils include a band-, bean-, or round-shaped nonsegmented nucleus and a higher nucleus-to-cytoplasm ratio (Fig. 1G and H; Latimer et al. 2003; Stacy et al. 2022). To our knowledge, our study is the first to quantify neutrophil precursors and toxic change in a wild amphibian.

Trypanosomes (Fig. 1I–L) were identified using morphological descriptions (Davis and Hopkins 2013) that have been used previously (DuRant et al. 2015; Hopkins et al. 2016; Bodinof Jachowski et al. 2024). We used the combined total of trypanosomes counted on both of the duplicate buffy coat smears to represent parasite relative abundance. Before the study started, we confirmed the reliability and reproducibility of the observer by blindly quantifying 20 whole blood smears in triplicate. We then used the intraclass correlation coefficient (ICC; Sokal and Rohlf 1995) and calculated the ICC score for leukocytes and trypanosomes (ICC neutrophils = 0.93; ICC lymphocytes = 0.95; ICC eosinophils = 0.92; ICC basophils = 0.85; ICC monocytes = 0.88; ICC trypanosomes = 0.98) to demonstrate a high degree of reproducibility. Our method of measuring trypanosome relative abundance is semiquantitative and has yet to be validated with quantitative Polymerase Chain Reaction (qPCR), so we also calculated the ICC score between the two duplicate buffy coat smears to confirm that our semiquantitative metric is consistently repeatable (ICC [duplicates] trypanosomes = 0.92).

### Nest fate

We determined ultimate nest outcomes following methods outlined in Hopkins et al. (2023). We considered nests to have failed if they no longer contained eggs or larvae. Conversely, we considered a nest to be successful if at least one larva was observed in the nest in the spring and the attending male was present during at least three of the four recapture attempts across the 8-month paternal care period. We determined whether nest failure was due to whole-clutch cannibalism if males were observed regurgitating eggs during capture or at least two of the following characteristics were observed: the clutch was torn apart with eggs missing, extreme bloating (or an obviously distended abdomen), or significant, abnormal weight gain since the prior capture (diagnostic characteristics described in detail in Hopkins et al. 2023).

### Statistical analysis

We used R (R Core Team 2022) to conduct all statistical analyses. Prior to analysis, we used the Shapiro–Wilk test for normality and applied a square root transformation on the proportion of eosinophils, basophils, band neutrophils, and toxic neutrophils, and then scaled all WBC variables to have a mean of 0 and a standard deviation (SD) of 1. Given our WBC variables were quantified as proportions, we chose a multivariate approach to reduce the dimensionality of the dataset and account for their interdependence. We performed a principal component analysis (PCA) based on a correlation matrix to retain the first principal component (PC) as a proxy of the proportion of total neutrophils (the sum of normal, band, and toxic neutrophils), lymphocytes, eosinophils, and basophils. On average, the proportion of monocytes did not exceed 2%, so they were excluded from the PCA and are not discussed further.

The abundance of leeches, leech bites, and trypanosomes varied widely among individuals, so we scaled each variable representing relative parasite abundance to have a mean of 0 and an SD of 1 (Schielzeth 2010) prior to using them as predictors in the models. Additionally, predictors related to the potential interactive effects of leeches and trypanosomes on the host and the interaction between parasite infection status and temperature on immune indices were included in the analysis.

Sampling day (oviposition/mid-embryonic development/hatching/emergence) and 24-h median temperature (°C) prior to capture were strongly confounded predictors. Thus, both variables could not be included within the same model. However, they did not produce consistently comparable model outputs. To address this, we performed preliminary model comparisons for all WBC response variables to determine which predictor was the best fit for each analysis. We found that temperature overwhelmingly outcompeted sampling day for all WBC variables except for the proportion of toxic neutrophils. Based on those results, we chose to use the 24-h median temperature (°C) as a continuous variable to represent seasonal effects when analyzing PCs and band neutrophils, while including sampling day as a categorical predictor for toxic neutrophils. We used the median temperature that hellbenders experienced 24 h prior to sampling for simplicity and because preliminary models indicated that it performed similarly to shorter and longer intervals.

We created linear mixed-effects models to assess how immune indices (i.e., PC scores, band neutrophils, and toxic neutrophils) responded to parasites and seasonal changes in water temperature. PC scores of WBCs were used as response variables, while predictors included water temperature and parasite abundance as continuous variables, and infection status as a categorical variable. Infection status represented whether hellbenders were uninfected, coinfected, or only infected with trypanosomes (we found no individuals only infected with leeches, see results). However, we also considered whether the effect of parasites depended on the severity of infection, which is why we also used parasite abundance in our initial model selection. However, we did not include both infection variables in the same models. We also included interactions between the abundance of leeches and trypanosomes as well as the interaction between infection status and temperature. Additionally, we performed separate analyses using the transformed and scaled values of band neutrophils and toxic neutrophils as response variables. We used the same predictors and interactions for these models but used sampling day instead of temperature in the model for toxic neutrophils because it outperformed temperature in preliminary models. We used the lme4 package for analyzing the PCs (WBC PC1 and PC2), band neutrophils, and toxic neutrophils with a normal distribution and identity link function. The individual PIT-tag ID was included as a random effect in all linear mixed-effects models to account for repeated measures on the same individuals.

We used generalized linear models to assess whether parasitic infection influenced nest outcomes. Prior research found that the majority of instances of whole-clutch cannibalism and nest failure can be confirmed within the first 2 months of embryonic development, prior to egg hatching (Hopkins et al. 2023). Thus, we used parasite infection status or parasite abundance at oviposition in our models to determine whether the severity of infection directly following nest establishment could predict ultimate nest outcomes. Using infection data at mid-embryonic development produced comparable results (analysis not shown). We performed two separate analyses and used the binary outcomes of nest success/nest failure and nest success/complete clutch cannibalism as the response variables. We used the glm function in the base package for analyzing nest outcomes with a binomial distribution. For our predictors, we included the abundance of leeches, leech bites, and trypanosomes as well as infection status. We also included interactions between the abundance of leeches and the abundance of trypanosomes. We then used Akaike's information criterion corrected for small sample sizes (AICc) (Burnham and Anderson 2002) and determined the best-fitting model by evaluating whether the delta AICc was greater than 2.

## Results

Over the 2 years of the study and multiple recaptures across the parental care period, we had 172 capture events and collected data from 58 individual adult male hellbenders with nests. We obtained a complete dataset including morphometrics, WBC profiles, and metrics to evaluate parasitic infection (leeches, leech bites, and trypanosomes) for 56 out of 58 individuals (Tables 1 and 2), and partial data for the remaining two individuals. Of the 58 nesting males, 25 were successful and 33 were failures from which 10 were confirmed whole-clutch cannibals. WBC profiles of hellbenders varied depending on cell type and sampling interval (Table 1) and all fell within the range previously documented for this species (Hopkins et al. 2016; Bodinof Jachowski et al. 2024) and expected of amphibians more broadly (Davis and Maerz 2023). Among the WBC differentials, lymphocytes were the most abundant cell type (median = 57%, range = 33–77%) followed by total neutrophils (normal, toxic, and band combined; median = 30%, range = 14–52%), eosinophils (median = 9%, range = 0.4–25%), basophils (median = 4%, range = 0–13%), and monocytes (median = 0.4%, range = 0–5%). Of their total WBC count, hellbenders also had considerable variation in the proportions of toxic (median = 15%, range = 3–40%) and band neutrophils (median = 5%, range = 0–27%). Likewise, we observed substantial variability in the abundance of leeches (range = 0–45), leech bites (range = 0–159), and trypanosomes (range = 0–323). Moreover, parasite prevalence and intensity of infection were variable across the paternal care period (Table 2).

**Table 2:** Characteristics of parasitic infection of adult male eastern hellbenders (*Cryptobranchus alleganiensis*) across the 8-month parental care period from a population in southwest Virginia, USA.

	Sampling day			
	Oviposition	Mid-embryonic	Hatching	Emergence
Parameter	*N* = 56	*N* = 48	*N* = 44	*N* = 25
Leech bites	15.3 ± 10.39	9.6 ± 2.66	9.0 ± 2.29	4.1 ± 1.24
Leech prevalence	42.8%	41.7%	30.2%	28.0%
Leech intensity	7.3 ± 1.98	1.8 ± 0.22	2.2 ± 0.45	1.7 ± 0.42
Trypanosome prevalence	83.9%	89.6%	88.4%	88.0%
Trypanosome intensity	64.0 ± 9.60	74.6 ± 9.15	57.7 ± 10.29	42.5 ± 7.10

Parasite prevalence represents the proportion of the population infected, and intensity is determined by the number of parasites present in an infected individual. Leech bites are represented as the arithmetic mean (±1 SEM).

**Fig. 2:**
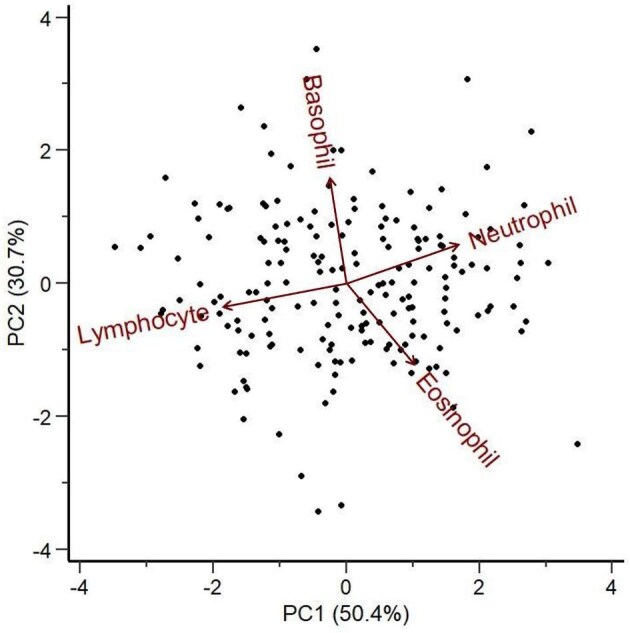
PCA biplot illustrating the relationships among WBC types in adult male hellbenders during the 8-month paternal care period. PC1 explained 50.4% of the variance and was positively loaded with neutrophils (0.616) and eosinophils (0.374) but negatively loaded with lymphocytes (−0.687). PC2 explained 30.7% of the variance and was positively loaded with basophils (0.749) and neutrophils (0.279) but negatively loaded with eosinophils (−0.578) and lymphocytes (−0.166). Arrows represent WBC loadings, with length indicating the strength of their influence, while points represent raw data points.

PC1 and PC2 explained 81% of the variance (Table 3). WBC PC1 explained 50.4% of the variance and was positively loaded with neutrophils and eosinophils, but was negatively loaded with lymphocytes (Table 3). Given the inverse relationship between neutrophils and lymphocytes found in PC1 (Supplemental Table 1), we consider the N:L ratio to be represented in this PC score. WBC PC2 explained 30.7% of the variance and was positively loaded with basophils and neutrophils but was negatively loaded with eosinophils and lymphocytes (Fig. 2). After evaluating the correlations between WBC profiles and PC2, we found that eosinophils and basophils had strong relationships with the PC scores while neutrophils and lymphocytes were not well represented in the PC2 scores (Supplemental Table 1).

**Table 3:** Results from PCA on the proportions of WBCs in male eastern hellbender (*Cryptobranchus alleganiensis*) blood and parameter loadings for each PC.

WBC PCA				
Parameters	PC1	PC2	PC3	PC4
Neutrophils	0.616	0.279	0.426	0.601
Lymphocytes	−0.687	−0.166	0.117	0.698
Eosinophils	0.374	−0.578	−0.641	0.339
Basophils		0.749	−0.627	0.192
Eigenvalue	2.016	1.229	0.744	0.011
Proportion of variance	0.504	0.307	0.186	0.003
Cumulative variance	0.504	0.811	0.997	1.000

**Fig. 3:**
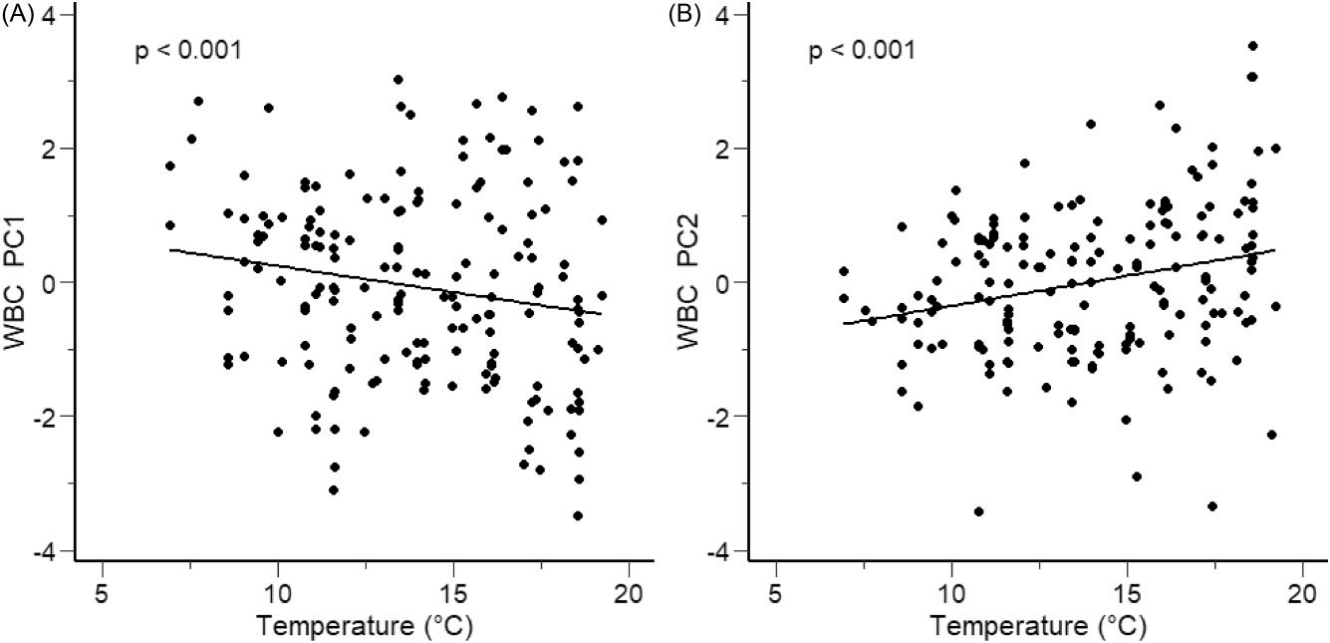
The relationship between temperature and the PC scores for WBC profiles in adult male hellbenders across the 8-month paternal care period. (**A**) The relationship between stream temperature (°C) and WBC PC1, which explained 50.4% of the variance and was positively loaded with neutrophils (0.616) and eosinophils (0.374), but was negatively loaded with lymphocytes (−0.687) (**B**) The relationship between stream temperature (°C) on WBC PC2, which explained 30.7% of the variance and was positively loaded with neutrophils (0.279) and basophils (0.749) but was negatively loaded with lymphocytes (−0.166) and eosinophils (−0.587). Lines represent model predictions from the top-ranking models using AICc, while points represent PC scores.

**Fig. 4:**
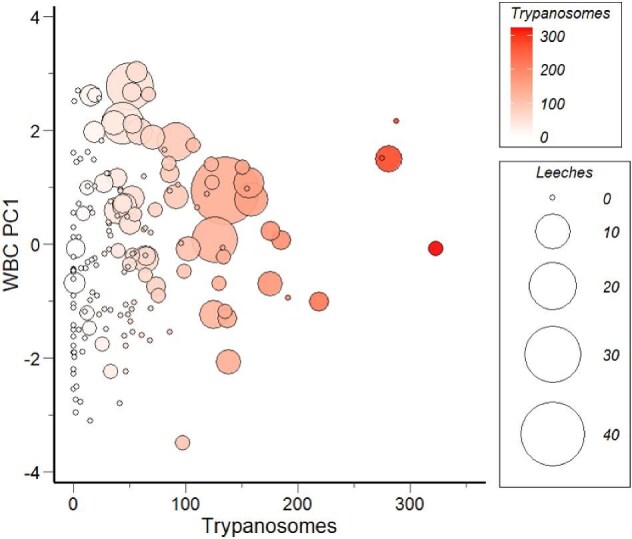
The relationship between the abundance of parasites and WBC PC1 in hellbenders, demonstrating the interactive effects between leeches and trypanosomes. Trypanosomes represent the total number of trypanosomes counted within 50 random fields of view across duplicate buffy coat slides while leeches represent the number of leeches attached to the individual at the time of capture. Corresponding to our results from the top-ranked model using AICc, as the size of the circles (leech abundance) increases, PC1 values exhibit a positive shift. However, as the size of the circles increases and increases in color (trypanosome abundance), PC1 values exhibit a negative shift.

Among the candidate models for WBC PC1, the top-ranking model representing the effects of water temperature and parasitic infection accumulated 0.63 of the AICc weight (Table 4). The delta AICc between the first- and second-ranked models was greater than 2, indicating that the top-ranked model was more likely to explain the variation in PC1 than the second-ranked model. As water temperature increased, PC1 decreased (β = −0.095, standard error [SE] = 0.028, *t* = −3.397, *P* < 0.001) (Fig. 3A). Considering the correlations between the PC scores and WBC profiles (Supplemental Table 1), we interpret this statistical outcome to signify that the proportion of lymphocytes in circulation was greatest during warm temperatures. In contrast, the proportions of neutrophils and eosinophils were lowest during warm temperatures. Post hoc visualization of the raw cell differentials against temperature confirmed these patterns (Supplemental Fig. 1). WBC PC1 was positively influenced by the number of leeches attached (β = 0.708, SE = 0.167, *t* = 4.243, *P* < 0.001). While trypanosome abundance alone did not significantly influence WBC PC1 (β = 0.099, SE = 0.108, *t* = 0.914, *P* = 0.362), as the abundance of trypanosomes and leeches increased together in coinfected individuals, there was a negative effect on PC1 (β = −0.403, SE = 0.137, *t* = −2.940, *P* = 0.004) (Fig. 4). The positive influence of leeches on PC1 values indicates that leech infection resulted in elevated neutrophils and eosinophils and lower lymphocytes. Interestingly, as the abundance of both leeches and trypanosomes increased in coinfected individuals, a negative shift occurred in PC1 values, indicating a greater number of lymphocytes in circulation and fewer eosinophils and neutrophils (Fig. 4).

**Table 4:** Results from linear mixed-effects model selection examining the effects of seasonal variation in temperature and parasites on WBC parameters in eastern hellbenders.

Linear mixed-effects models	K	AICc	Delta AICc	AICc weight	Cumulative weight
PC1 ∼ temperature + trypanosomes × leeches	7	578.78	0	0.63	0.63
PC1 ∼ temperature + trypanosomes × leeches + leech bites	8	581.76	2.98	0.14	0.77
PC1 ∼ temperature + trypanosomes + leeches	6	582.61	3.82	0.09	0.87
PC1 ∼ 1	3	582.97	4.18	0.08	0.94
PC1 ∼ temperature	4	584.52	5.74	0.04	0.98
PC1 ∼ temperature + trypanosomes	5	586.97	8.18	0.01	0.99
PC1 ∼ temperature + trypanosomes + leeches + leech bites	7	587.01	8.23	0.01	1.00
PC1 ∼ infection status × temperature	8	592.84	14.06	0.00	1.00

AICc indicates Akaike’s information criterion corrected for small sample size; delta AICc is a measure of each model relative to the model with the smallest AICc; AICc weight is the relative likelihood of a model, normalized across all candidate models; cumulative weight is the cumulative sum of AICc weights as models are ranked; K represents the number of parameters in the model, including fixed effects, random effects, and the intercept.

*Note*: All models included individual ID as a random effect.

The top-ranked model for WBC PC2, which only included temperature as a predictor, had an AICc weight of 0.83 and a delta AIC greater than 2, confirming it outcompeted the lower-ranking candidate models (Table 5). WBC PC2 increased as temperature increased (β = 0.100, SE = 0.022, *t* = 4.511, *P* < 0.001) (Fig. 3B). When considering the relationship between WBC profiles and PC2 (Supplemental Table 1), we interpret our model to indicate that as temperature increased, basophils increased while eosinophils decreased in circulation. Post hoc visualization of the raw cell differentials against temperature confirmed these patterns (Supplemental Fig. 1).

**Table 5:** Results from linear mixed-effects model selection examining the effects of seasonal variation in temperature and parasites on WBC parameters in eastern hellbenders.

Linear mixed-effects models	K	AICc	Delta AICc	AICc weight	Cumulative weight
PC2 ∼ temperature	4	510.06	0.00	0.83	0.83
PC2 ∼ temperature + trypanosomes	5	513.59	3.53	0.14	0.97
PC2 ∼ temperature + trypanosomes + leeches	6	518.55	8.49	0.01	0.98
PC2 ∼ temperature + trypanosomes × leeches	7	518.78	8.72	0.01	0.99
PC2 ∼ infection status × temperature	8	520.30	10.42	0.00	0.99
PC2 ∼ 1	3	521.25	11.19	0.00	1.00
PC2 ∼ temperature + trypanosomes × leeches + leech bites	8	523.32	13.25	0.00	1.00
PC2 ∼ temperature + trypanosomes + leeches + leech bites	7	523.40	13.33	0.00	1.00

AICc indicates Akaike’s information criterion corrected for small sample size; delta AICc is a measure of each model relative to the model with the smallest AICc; AICc weight is the relative likelihood of a model, normalized across all candidate models; cumulative weight is the cumulative sum of AICc weights as models are ranked; K represents the number of parameters in the model, including fixed effects, random effects, and the intercept.

*Note*: All models included individual ID as a random effect.

**Fig. 5:**
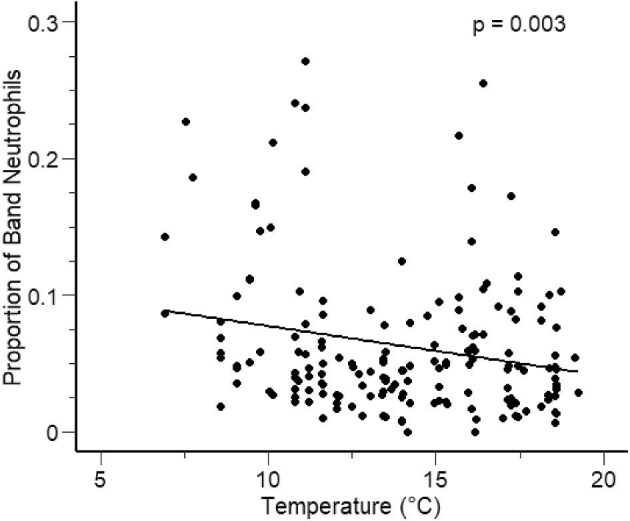
The relationship between temperature and the proportion of band neutrophils in adult male hellbenders across the 8-month paternal care period. The line represents model predictions from the top ranking model using AICc, while the points represent observed values.

The top-ranked model for band neutrophils also only included temperature as a predictor and had an AICc weight of 0.55. However, the second-ranked model was the null model, which had a weight of 0.35, and the delta AICc between the two models was less than 2 (Table 6), indicating that temperature provided a modest improvement in model fit compared to the null model, but there remains considerable variation in the data that our more complex models were unable to explain. The proportion of band neutrophils increased in circulation as temperature decreased (β = −0.065, SE = 0.022, *t* = −2.999, *P* = 0.003) (Fig. 5).

**Table 6:** Results from linear mixed-effects model selection examining the effects of seasonal variation in temperature and parasites on band neutrophils in eastern hellbenders.

Linear mixed-effects models	K	AICc	Delta AICc	AICc weight	Cumulative weight
Band neutrophils ∼ temperature	4	483.83	0.00	0.55	0.55
Band neutrophils ∼ 1	3	484.71	0.88	0.35	0.90
Band neutrophils ∼ temperature + trypanosomes	5	489.01	5.18	0.04	0.95
Band neutrophils ∼ temperature + leeches × trypanosomes	7	489.44	5.60	0.03	0.98
Band neutrophils ∼ temperature + leeches × trypanosomes + leech bites	8	490.91	7.07	0.02	1.00
Band neutrophils ∼ temperature + leeches + trypanosomes	6	493.48	9.64	0.00	1.00
Band neutrophils ∼ temperature + leeches + trypanosomes + leech bites	7	497.90	14.06	0.00	1.00
Band neutrophils ∼ infection status × temperature	8	502.34	18.51	0.00	1.00

AICc indicates Akaike’s information criterion corrected for small sample size; delta AICc is a measure of each model relative to the model with the smallest AICc; AICc weight is the relative likelihood of a model, normalized across all candidate models; cumulative weight is the cumulative sum of AICc weights as models are ranked; K represents the number of parameters in the model, including fixed effects, random effects, and the intercept.

*Note*: All models included individual ID as a random effect.

To qualitatively summarize the effects of water temperature on various cell types, we found that as temperatures increased from ∼5°C to 20°C, the proportion (on average) of neutrophils decreased by 14% and the proportion of eosinophils decreased by 46%. Conversely, the proportion (on average) of lymphocytes increased by 8% and the proportion of basophils increased by 101%. Lastly, the proportion of band neutrophils decreased by 80% (Supplemental Fig. 1).

**Fig. 6:**
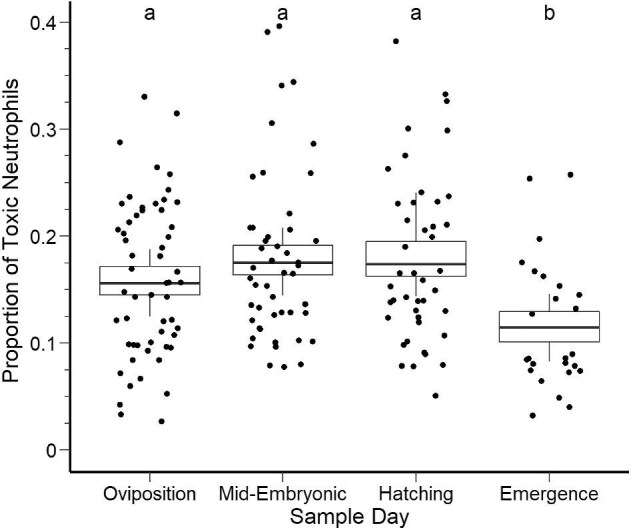
Seasonal differences in the proportion of toxic neutrophils across the 8-month parental care period of hellbenders. Box plots represent the distribution of model predictions from the top-ranking models using AICc while the points represent raw data. Letters in superscript indicate statistical differences in the proportion of toxic neutrophils across the parental care period (Supplemental Table 2). Sampling day represents repeated capture intervals corresponding to offspring development in which oviposition represents nest initiation (Day ∼0), mid-embryonic represents mid-embryonic development (Day ∼30), hatching represents larval hatching (Day ∼60), and emergence represents spring larval emergence (Day ∼200).

Unlike models for other cell types, the top-ranked model for toxic neutrophils only included sample day (rather than temperature) as a predictor and had an AICc weight of 0.86 with a delta AIC greater than 2, confirming it outcompeted the lower-ranking candidate models (Table 7). Compared to values during oviposition, the proportion of toxic neutrophils did not significantly differ at mid-embryonic development (β = 0.271, SE = 0.173, *t* = 1.570, *P* = 0.119) or at hatching (β = 0.254, SE = 0.179, *t* = 1.417, *P* = 0.159). However, the proportion of toxic neutrophils was significantly lower at spring emergence compared to the rest of the parental care sampling periods (β = −0.574, SE = 0.213, *t* = −2.688, *P* = 0.008) (Fig. 6; Supplemental Table 2). For the candidate models predicting nest success, the null was the top-ranking model with an AICc weight of 0.26. The following three candidate models accumulated similar AICc weights. The low delta AICc values (Supplemental Table 3) indicate that there was considerable uncertainty in the model selection and suggest that the predictors representing parasite infection are unlikely to explain the likelihood of nest success.

**Table 7:** Results from linear-mixed effects model selection examining the effects of seasonal variation and parasites on toxic neutrophils in eastern hellbenders.

Linear mixed-effects models	K	AICc	Delta AICc	AICc weight	Cumulative weight
Toxic neutrophils ∼ sample day	6	477.29	0.00	0.86	0.86
Toxic neutrophils ∼ sample day + trypanosomes	7	482.04	4.75	0.08	0.94
Toxic neutrophils ∼ 1	3	483.04	5.75	0.05	0.99
Toxic neutrophils ∼ sample day + trypanosomes + leeches	8	487.53	10.24	0.01	0.99
Toxic neutrophils ∼ sample day + trypanosomes + leeches + leech bites	9	489.90	12.61	0.00	1.00
Toxic neutrophils ∼ sample day × infection status	14	490.97	13.68	0.00	1.00
Toxic neutrophils ∼ sample day + trypanosomes × leeches	9	492.40	15.11	0.00	1.00
Toxic neutrophils ∼ sample day + trypanosomes × leeches + leech bites	10	494.44	17.15	0.00	1.00

AICc indicates Akaike’s information criterion corrected for small sample size; delta AICc is a measure of each model relative to the model with the smallest AICc; AICc weight is the relative likelihood of a model, normalized across all candidate models; cumulative weight is the cumulative sum of AICc weights as models are ranked; K represents the number of parameters in the model, including fixed effects, random effects, and the intercept.

*Note*: All models included individual ID as a random effect.

**Fig. 7:**
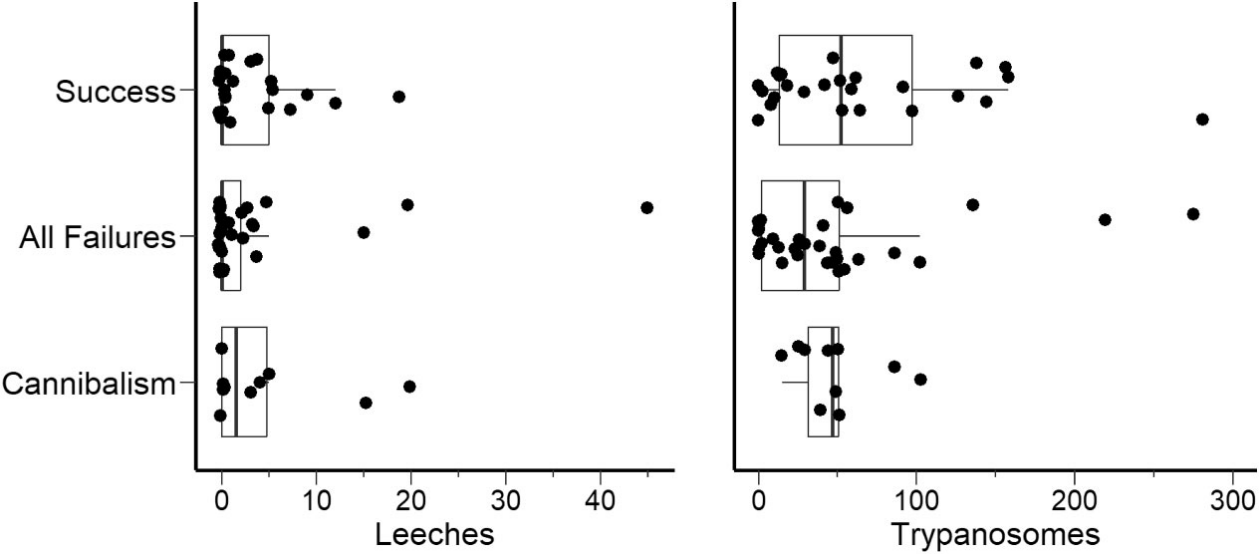
The relationship between parasitic infection and fate of hellbenders nests. (**A**) The distribution of nest outcomes in response to the abundance of leeches during oviposition. (**B**) The distribution of nest outcomes in response to the abundance of trypanosomes during oviposition. Box plots represent the relative distribution of parasite abundance within the categorized nest outcome and points indicate raw data. Trypanosome abundance represents the total number of trypanosomes observed within 50 random fields of view on buffy coat smears. Cannibalism represents the nest fate category in which nest failure was confirmed as whole-clutch cannibalism. While all categories of nest outcomes are expressed in the figure, statistical analyses were performed separately to evaluate whether parasite infection influenced the likelihood of success versus failure and success versus whole-clutch cannibalism.

For the candidate models predicting the likelihood of whole-clutch cannibalism, the top-ranking model included the abundance of parasites and the interaction between the leeches and trypanosomes as the predictors, which had an AICc weight of 0.54. The second-ranked model was the null with a weight of 0.20 and a delta AICc of less than 2 (Supplemental Table 4), indicating that predictors included in the top model might be relevant but did not substantially improve the overall model fit. The likelihood of whole-clutch cannibalism was not significantly influenced by the abundance of trypanosomes (β = −0.954, SE = 0.981, *z* = −0.972, *P* = 0.331), leeches (β = 1.230, SE = 1.046, z = 1.176, *P* = 0.240), or the interaction between the two parasites (β = −4.140, SE = 2.500, *z* = −1.656, *P* = 0.098). Considering the results from both analyses exploring nest fate, we found no evidence to indicate that parasite infection influences the likelihood of nest success or whole-clutch cannibalism (Fig. 7A and B).

## Discussion

Parasites and pathogens contribute to global amphibian declines by impacting host survival and fitness-related traits, but parasites that are not directly linked to high morbidities or mortality in amphibians draw comparably less attention than these more dramatic examples (Bower et al. 2019). Additionally, infectious diseases tend to cause greater impacts on small populations of threatened species than more abundant species (May 1988), emphasizing the need to understand the sublethal effects of parasites in species of conservation concern. To effectively do so in wild amphibian populations, however, it is crucial to understand how immune indices shift with environmental conditions to distinguish pathological effects from natural variation. In this study, we used WBC profiles to assess the health and immune responses of male eastern hellbender salamanders to parasitic infections at varying temperatures throughout their ∼8-month paternal care period. Our objectives were to evaluate the impacts of parasites and coinfections on host physiology and to determine whether parasitic infection negatively affects nest success. We demonstrated that reference values for WBC profiles of hellbenders from healthy populations vary with seasonal temperatures. We found that coinfection of leeches and trypanosomes is associated with immune responses in hellbenders, but we found no evidence to suggest that parasite infection or coinfection affects nest success.

We observed changes in hellbender WBC profiles that correlated with seasonal fluctuations in environmental temperatures. As predicted, the proportion of circulating basophils and lymphocytes was highest during warmer periods of the paternal care period. Basophils are considered short-lived cells and migrate out of circulation and into tissues upon activation (Eberle and Voehringer 2016) similar to how lymphocytes typically respond (Latimer et al. 2003). The seasonal dynamics of basophil proliferation in amphibians, however, remain underexplored in the literature and our identification of a correlation between temperature and circulating basophils represents a novel contribution to our understanding of amphibian immunology. In contrast to basophils, hellbenders had lower proportions of circulating neutrophils and eosinophils in warmer periods of paternal care. Similar fluctuations in WBCs in response to temperature have been observed in other amphibians (Maniero and Carey 1997; Raffel et al. 2006b). Our findings also align with prior research on hellbenders where N:L ratios and eosinophils were lower in warmer compared to colder temperatures (Bodinof Jachowski et al. 2024).

Although cold temperatures can exert immunosuppressive effects on some amphibians (Rollins-Smith and Woodhams 2012; Herczeg et al. 2021), our study highlights the potential for physiological adaptations to cold conditions, which may serve to prime the cellular immune system when disease susceptibility is heightened (Greenspan et al. 2017). Characteristics of the adaptive immune system in ectotherms, such as lymphocyte production, antibody responses, and complement activity, are reduced in cold temperatures, likely because they are metabolically expensive (Rijkers et al. 1980; Cooper et al. 1992; Maniero and Carey 1997; Rollins-Smith and Woodhams 2012). To overcome this, ectotherms rely on elements of their innate immune responses to reinforce their immune defenses by increasing circulating neutrophils (Maniero and Carey 1997; Raffel et al. 2006b) and phagocytic activity (Pxytycz and Jozkowicz 1994). Considering the phagocytic capabilities of neutrophils, and to a lesser extent, eosinophils (Latimer et al. 2003), the observed increase in the proportion of circulating neutrophils and eosinophils in colder temperatures could be an adaptative strategy that protects hellbenders when other immune defenses are less active (Maniero and Carey 1997), or to prepare them for an increased risk of encountering pathogens in spring (Pxytycz and Jozkowicz 1994).

The significance of neutrophils in seasonal protective immunity is further emphasized by our identification of seasonal shifts in the morphological characteristics of neutrophils, signifying seasonal differences in immune cell recruitment. Toxic neutrophils are mature cells that exhibit toxic changes due to accelerated production, causing abnormal maturation, while band neutrophils are immature cells that increase in circulation when immune demand exceeds neutrophil storage pools (Stacy et al. 2022). The proportion of band neutrophils in circulation increased as temperature decreased, which has been observed in another amphibian species in response to long-term laboratory exposure to low temperatures (Maniero and Carey 1997). Moreover, we found that the proportion of toxic neutrophils in circulation remained the same across oviposition, mid-embryonic development, and hatching but significantly decreased during larval emergence. This observation suggests that rapid demand for circulating neutrophils subsides in the spring as water is warming. When considering the concurrent trends of both band and toxic neutrophils, our results imply a prolonged demand for neutrophils across the paternal care period, with a notable increase during the coldest period prior to overwintering, that may serve to maintain immunocompetence throughout the year. Taken together, these results underscore the possible critical role of neutrophils in seasonal protective immunity in hellbenders and suggest further work is needed to understand their seasonal immunological role in other amphibian species.

It is plausible that factors other than temperature may have contributed to some of the seasonal effects that we observed, and the nature of our descriptive field study prevents us from identifying possible contributions of other seasonal factors. Raffel et al. (2006b) suggested that there may be seasonal variations in circulating WBCs that are independent of temperature. Our findings on toxic neutrophils underscore a seasonal trend that supports this possibility. Furthermore, seasonal fluctuations in behavior and physiology linked to reproduction could impact immune indices at different times of the year. For example, circannual rhythms in hormones such as androgens and glucocorticoids, known to modulate immune function, might contribute to seasonal variations in WBCs (Zapata et al. 1992). Notably, seasonal variations in these hormones have been observed in male hellbenders (Case et al. 2024), providing support for this possibility. Future experimental work is required to disentangle the relative influence of temperature and other seasonally variable factors on WBCs.

Hellbenders exhibited notable shifts in WBC profiles in response to parasitic infection, but this response depended on which parasites infected the host. As predicted, when the abundance of leeches increased, the proportion of neutrophils and eosinophils increased, and lymphocytes decreased. These patterns were similar to those observed in two prior studies when samples were collected in the summer prior to the breeding season (DuRant et al. 2015; Hopkins et al. 2016). Our findings also suggest the likelihood that parasites caused the increase in N:L ratios and eosinophils compared to uninfected individuals observed in a previous landscape-scale study, which could not disentangle the effects of habitat quality from parasitic infection because they were confounded (Bodinof Jachowski et al. 2024). By focusing on a narrow range of habitat quality in a single stream in our study, we were able to isolate the impact of parasites on WBC counts from other habitat-related factors. In contrast to the results for leeches, we did not find evidence that trypanosomes alone altered WBC profiles. Our results suggest that in the absence of leech infection, trypanosome infection was not associated with a strong immune response in hellbenders, regardless of infection intensity. This could be because most aquatic trypanosomes are nonpathogenic to fish (Woo 1994, 2006) and amphibian hosts (Omonona and Ekpenko 2011). Alternatively, other species of trypanosomes have well-documented strategies for evading the hosts’ immune responses (Oladrian and Belosevic 2012), which is an equally plausible explanation. In notable contrast to our findings, Hopkins et al. (2016) found a strong relationship between trypanosome presence and increased circulating eosinophils and N:L ratios in hellbenders. The difference between our findings and theirs could be attributed to the method used to represent trypanosome infections. Whereas we used a semi-quantitative measure of parasite abundance to assess the severity of infection, they used a presence/absence metric to indicate the incidence of infection. However, our metric of trypanosome infection severity could be even more useful if validated against qPCR in future studies.

We detected an unexpected interaction between leeches and trypanosomes in coinfected hellbenders. That is, as the abundance of both leeches and trypanosomes increased concurrently, the proportion of lymphocytes increased in circulation while eosinophils and neutrophils decreased. In other host–parasite systems, the temporal sequence of parasite exposure can strongly influence the host's response and disease progression (Herczeg et al. 2021), which might explain the difference in WBC profiles when evaluating the effect of leech abundance alone versus the interaction between leech and trypanosome abundance. Leeches, acting as vectors for trypanosomes in hellbenders, engage in active parental care behaviors, delivering their offspring to their first blood meal (A. Blumenthal and W. A. Hopkins, unpublished). Consequently, hellbender hosts likely mount an immune response to multiple biting leech vectors during the initial transmission of the trypanosome parasites. Repeated exposure to salivary antigens from leeches may prompt the host to develop cellular and humoral reactions, altering the site of leech attachment, which may promote rejection of the ectoparasite (Wikel 1982). Leeches have been linked to other immunological responses in hellbenders indicative of inflammation and infection such as increased total plasma proteins as well as increased bactericidal capacity of plasma (Hopkins et al. 2016). While elevated eosinophils, in particular, are often an indicator of parasitic disease states (Allender and Fry 2008; Bain and Harr 2022), neutrophils and eosinophils are recruited into circulation in response to inflammation and infection (Latimer et al. 2003; Campbell 2015). Thus, the observed shifts in neutrophils, eosinophils, and lymphocytes potentially indicate an active innate immune response to leeches, recruiting inflammatory cells to the site of ectoparasite exposure (Andrade et al. 2005). After the initial leech infection, trypanosomes likely proliferate within coinfected hellbenders and the host's immune response undergoes a shift, which we hypothesize contributes to the observed interaction with leeches. The reduction in neutrophils and eosinophils might suggest an attempt to mitigate the inflammatory response influenced by the leech vector, possibly to prevent excessive tissue damage. Alternatively, it could indicate immune exhaustion where the emigration of neutrophils and eosinophils from the vasculature to the tissues exceeds the replacement rate in the blood (Latimer et al. 2003) and could be a sign of severe, acute inflammatory disease (Bain and Harr 2022). Lymphocytes are the primary cells in the host's adaptive immune response to infection (Janeway et al. 2001) and perform a variety of immune functions, including immunoglobin production and antibody-dependent cell-mediated cytotoxicity (Wright and Whitaker 2001; Cambell 2015). Thus, the observed increase in circulating lymphocytes might suggest acquired immunity as the severity of leech and trypanosome infection increases concurrently. In mammalian systems that have been studied more extensively, hosts rely on B and T lymphocytes for protection against trypanosomes and eventual elimination of the parasites, with B cells particularly responsible for clearing the parasites from blood during infection (Mansfield and Paulnock 2005). Therefore, coinfected hellbender hosts may exhibit a similar immune response to trypanosome infection, though it is unclear as to why this response would depend on the presence of the leech vector. Our reliance on WBC differentials alone limited our ability to draw conclusions regarding the observed immunological patterns, but future studies that include complete WBC counts and cytokines could enhance our understanding of the underlying mechanism.

Contrary to our predictions, we did not identify relationships between parasite infection and basophils, band neutrophils, or toxic neutrophils. Basophils offer protective immunity against both ecto- and endoparasites (Eberle and Voehringer 2016; Karasuyama et al. 2021) and may play a surveillance role by recruiting eosinophils during parasite infection as they do in other vertebrates (Wright and Whitaker 2001; Campbell 2015). Basophils are short-lived cells that migrate out of circulation when activated (Eberle and Voehringer 2016), but the proportion of circulating basophils differs among species (Megarani et al. 2020; Davis and Maerz 2023). Because basophils make up a relatively small proportion of circulating WBCs in hellbenders (Hopkins et al. 2016; Bodinof Jachowski et al. 2024), we may have failed to capture sufficient variation in basophil proportions for a detectable response to parasite infection against the backdrop of substantial seasonal variation in basophils. Similarly, we were unable to directly link elevated band and toxic neutrophils to parasitic infection, possibly due to a lag time in immune responses. For example, Slack et al. (2024) demonstrated that coinfected hellbenders do not exhibit symptoms of regenerative anemia until ∼30 days following peak leech attachment. Additionally, hellbenders may exhibit signs of immune recruitment in response to other infectious pathogens that we did not evaluate; leeches may transmit viruses, fungi, and bacteria to hellbenders, as they do in other systems (Sawyer 1986; Mock 1987; Barta and Desser 1989; Raffel et al. 2006a). Toxic neutrophils are known to occur as part of the immune response of amphibians to Gram‐negative bacteria found in aquatic environments, such as *Vibrio* and *Aeromonas* (Bain and Harr 2022). We encourage future research to determine whether leeches transmit other pathogens to hellbenders and whether this has the potential to contribute to some of the immune responses that we observed. Use of qPCR to identify the possible influence of other co-occurring pathogens could strengthen inferences about causative relationships between parasites and immune responses.

While parasitic coinfection influenced hellbender physiology, we found no evidence relating parasites to nest failure or whole-clutch filial cannibalism. In other systems, parasites can modulate the behavior and metabolic demands of the host (Haye and Ojeda 1998; Dick et al. 2010) even leading to increased rates of cannibalism (Bunke et al. 2015). Additionally, parasites can reduce mating success, fecundity, and offspring viability in other hosts (Lehmann 1993; Hasik and Siepielski 2022). While our research found no evidence to indicate that parasites are a direct catalyst for increased cannibalism or nest failure in hellbenders, the impacts of infection on other fitness-related traits and behaviors are complex and worthy of future investigation. For example, parasite infection in other vertebrates is associated with reduced reproductive and paternal behaviors (Saumier et al. 1986; Henderson et al. 1995; but see Klein 2003). Given that we recently found coinfection with leeches and trypanosomes was associated with multiple symptoms of anemia in hellbenders (Slack et al. 2024), it seems possible that parasites could modulate a shift from paternal care (e.g., tail fanning of eggs; O'Brien et al. 2024) to self-maintenance behaviors in hellbenders (e.g., side-to-side rocking to increase skin oxygenation; O'Brien et al. 2024), which could affect hatching success of eggs. Therefore, future studies that simultaneously examine the effect of leech and trypanosome infection on red blood cell physiology, immune responses, and parental care behaviors would be valuable.

By investigating the impacts of parasitic infection and environmental conditions on hellbender immunology, our research offers valuable insights into the basic physiology of this imperiled species. Our findings reveal seasonal shifts in hellbender WBC profiles, including indicators of immune cell recruitment that may reflect immunological adaptations to fluctuating temperatures. Thus, our work provides valuable reference values for the species. Additionally, we demonstrated that coinfecting parasites influence hellbender physiology, but their immunological responses were complex and depended on infection intensity of both parasites. Future experimental research could help disentangle some of these complexities, and help isolate the effect of temperature from other seasonal factors that could affect hellbender immunology. More broadly, our research addresses fundamental knowledge gaps related to amphibian immunology and host–parasite interactions, with broader implications for understanding amphibian population declines in the Anthropocene.

## Supplementary Material

obaf006_Supplemental_Files

## Data Availability

The data underlying this article will be shared on reasonable request to the corresponding author.
