# Supplementary material for: Parasite Infections Influence Immunological Responses But Not Reproductive Success of Male Hellbender Salamanders (Cryptobranchus alleganiensis)
Source: Integr Org Biol. 2025 Apr 3;7(1):obaf006. doi: 10.1093/iob/obaf006 (PMC12004113; doi:10.1093/iob/obaf006)
Supplement: obaf006_Supplemental_Files [file obaf006_supplemental_files.zip › Supplemental_fig_1.docx]

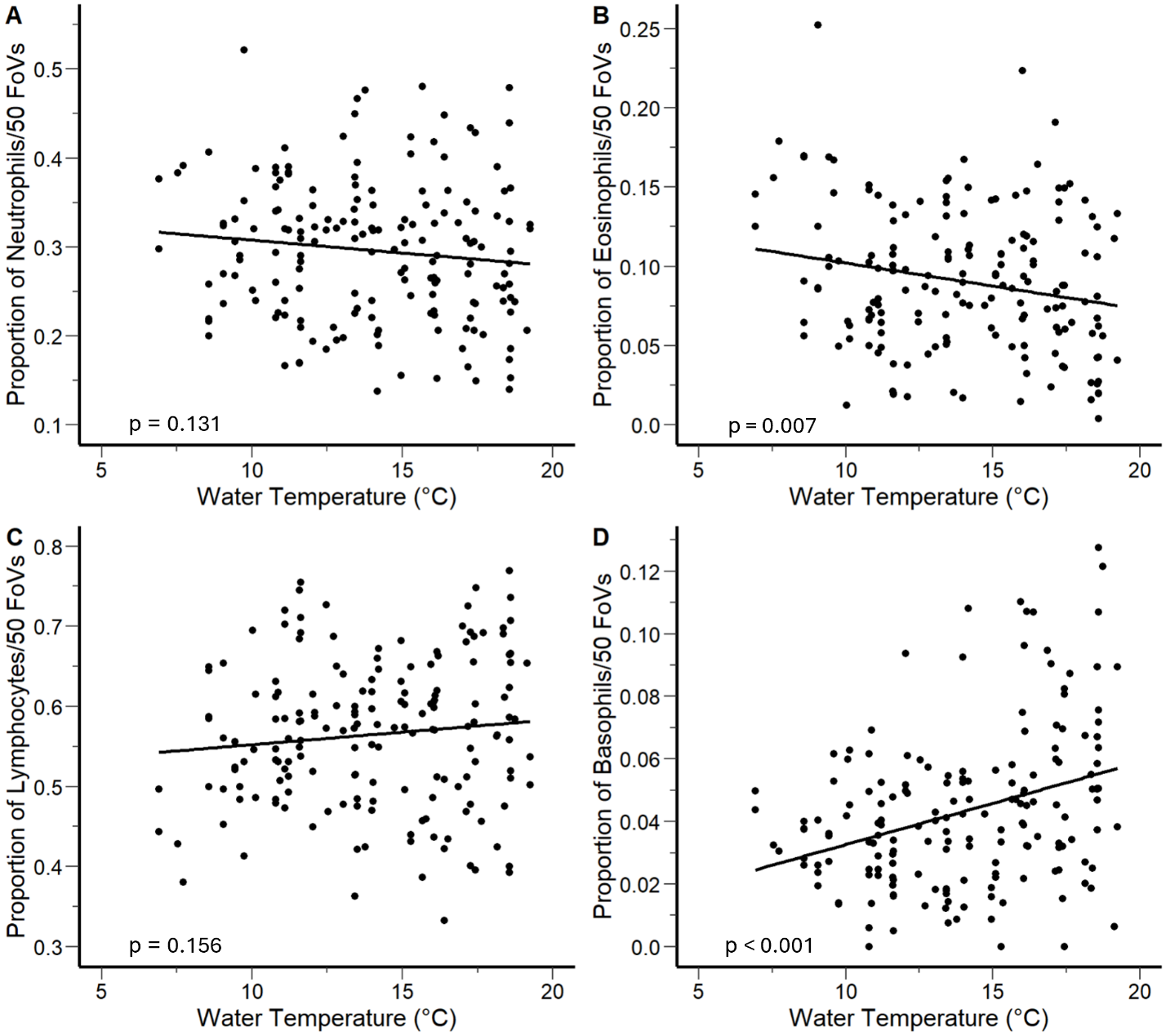


**Supplemental Figure 1.** The relationship between temperature and proportions of circulating white blood cells in male hellbenders across the 8-month parental care period. **(A)** The relationship between stream temperature (°C) and the proportion of neutrophils within 50 fields of view. **(B)** The relationship between stream temperature (°C) and the proportion of eosinophils within 50 fields of view. **(C)** The relationship between stream temperature (°C) and the proportion of lymphocytes within 50 fields of view. **(D)** The relationship between stream temperature (°C) and the proportion of basophils within 50 fields of view. Lines represent predictions from simple linear regressions from univariate models, while points represent raw data values. Note that this figure is for the purpose of qualitative visualization and does not reflect the statistical analysis that was performed.
