# Supplementary material for: Parasite Infections Influence Immunological Responses But Not Reproductive Success of Male Hellbender Salamanders (Cryptobranchus alleganiensis)
Source: Integr Org Biol. 2025 Apr 3;7(1):obaf006. doi: 10.1093/iob/obaf006 (PMC12004113; doi:10.1093/iob/obaf006)
Supplement: obaf006_Supplemental_Files [file obaf006_supplemental_files.zip › Supplemental_table_1.docx]

| **Supplemental Table 1** Pearson’s correlation coefficients between the proportion of white blood cells in male hellbenders and the resulting principal component scores. | | |
| --- | --- | --- |
| **Parameters** | **PC1** | **PC2** |
| Neutrophils | 0.875 | 0.309 |
| Lymphocytes | -0.975 | -0.184 |
| Eosinophils | 0.531 | -0.641 |
| Basophils | -0.131 | 0.830 |
