# Supplementary material for: Parasite Infections Influence Immunological Responses But Not Reproductive Success of Male Hellbender Salamanders (Cryptobranchus alleganiensis)
Source: Integr Org Biol. 2025 Apr 3;7(1):obaf006. doi: 10.1093/iob/obaf006 (PMC12004113; doi:10.1093/iob/obaf006)
Supplement: obaf006_Supplemental_Files [file obaf006_supplemental_files.zip › Supplemental_table_2.docx]

| **Supplemental Table 2** Statistical outcomes from pairwise comparisons of toxic neutrophils between sampling days. | | | |
| --- | --- | --- | --- |
| **Contrast** | **Estimate** | **SE** | **p value** |
| Oviposition / Mid-Embryonic | -0.271 | 0.173 | 0.400 |
| Oviposition / Hatching | -0.254 | 0.179 | 0.492 |
| Oviposition / Emergence | 0.574 | 0.214 | 0.041 |
| Mid-Embryonic / Hatching | 0.018 | 0.184 | 0.999 |
| Mid-Embryonic / Emergence | 0.845 | 0.219 | 0.001 |
| Hatching / Emergence | 0.828 | 0.223 | 0.002 |
| Statistical outcomes correspond to data shown in Figure 6. Estimates and standard error are on the response scale which was transformed, scaled, and centered prior to analysis. Sampling day represents repeated capture intervals corresponding to offspring development in which oviposition represents nest initiation [day ~0], mid-embryonic represents mid-embryonic development [day ~30], hatching represents larval hatching [day ~60], and emergence represents spring larval emergence [day ~200]. | | | |
