# Supplementary material for: Parasite Infections Influence Immunological Responses But Not Reproductive Success of Male Hellbender Salamanders (Cryptobranchus alleganiensis)
Source: Integr Org Biol. 2025 Apr 3;7(1):obaf006. doi: 10.1093/iob/obaf006 (PMC12004113; doi:10.1093/iob/obaf006)
Supplement: obaf006_Supplemental_Files [file obaf006_supplemental_files.zip › Supplemental_table_3.docx]

| **Supplemental Table 3** Results from generalized linear model selection examining the effects of parasites on nest fate (success vs. all failures) in eastern hellbenders. | | | | | |
| --- | --- | --- | --- | --- | --- |
| **Linear Mixed Effects Models** | **K** | **AICc** | **Delta AICc** | **AICc Weight** | **Cumulative Weight** |
| Success ~ 1 | 1 | 81.37 | 0.00 | 0.26 | 0.26 |
| Success ~ Trypanosomes | 2 | 81.94 | 0.57 | 0.19 | 0.45 |
| Success ~ Infection status + Leech bites | 4 | 82.03 | 0.66 | 0.19 | 0.64 |
| Success ~ Leech bites + Trypanosomes + Leeches | 4 | 82.28 | 0.91 | 0.16 | 0.80 |
| Success ~ Infection status | 3 | 83.55 | 2.18 | 0.09 | 0.89 |
| Success ~ Leeches + Trypanosomes | 3 | 83.83 | 2.46 | 0.08 | 0.96 |
| Success ~ Trypanosomes*Leeches | 4 | 85.28 | 3.91 | 0.04 | 1.00 |
| AICc indicates Akaike Information Criterion corrected for small sample size; Delta AICc is a measure of each model relative to the model with the smallest AICc; AICc Weight is the relative likelihood of a model, normalized across all candidate models; Cumulative Weight is the cumulative sum of AICc weights as models are ranked; K represents the number of parameters in the model. | | | | | |
