# Supplementary material for: Parasite Infections Influence Immunological Responses But Not Reproductive Success of Male Hellbender Salamanders (Cryptobranchus alleganiensis)
Source: Integr Org Biol. 2025 Apr 3;7(1):obaf006. doi: 10.1093/iob/obaf006 (PMC12004113; doi:10.1093/iob/obaf006)
Supplement: obaf006_Supplemental_Files [file obaf006_supplemental_files.zip › Supplemental_table_4.docx]

| **Supplemental Table 4** Results from generalized linear model selection examining the effects of parasites on nest failure resulting from whole clutch cannibalism in eastern hellbenders. | | | | | |
| --- | --- | --- | --- | --- | --- |
| **Linear Mixed Effects Models** | **K** | **AICc** | **Delta AICc** | **AICc Weight** | **Cumulative Weight** |
| Cannibal ~ Leeches*Trypanosomes | 4 | 42.05 | 0.00 | 0.54 | 0.54 |
| Cannibal ~ 1 | 1 | 44.00 | 1.95 | 0.20 | 0.75 |
| Cannibal ~ Trypanosomes | 2 | 45.51 | 3.46 | 0.10 | 0.84 |
| Cannibal ~ Leeches + Trypanosomes | 3 | 46.26 | 4.20 | 0.07 | 0.91 |
| Cannibal ~ Infection status | 3 | 47.25 | 5.19 | 0.04 | 0.95 |
| Cannibal ~ Leech bites + Trypanosomes + Leeches | 4 | 48.12 | 6.07 | 0.03 | 0.97 |
| Cannibal ~ Infection status + Leech bites | 4 | 48.17 | 6.12 | 0.03 | 1.00 |
| AICc indicates Akaike Information Criterion corrected for small sample size; Delta AICc is a measure of each model relative to the model with the smallest AICc; AICc Weight is the relative likelihood of a model, normalized across all candidate models; Cumulative Weight is the cumulative sum of AICc weights as models are ranked; K represents the number of parameters in the model. | | | | | |
